# Supplementary material for: DOT1L safeguards cartilage homeostasis and protects against osteoarthritis
Source: Nat Commun. 2017 Jun 19;8:15889. doi: 10.1038/ncomms15889 (PMC5481839; doi:10.1038/ncomms15889)
Supplement: Supplementary Information [file ncomms15889-s1.pdf]

Type of file: PDF
Title of file for HTML: Supplementary Information
Description: Supplementary Figures and Supplementary Tables

Type of file: PDF
Title of file for HTML: Peer Review File

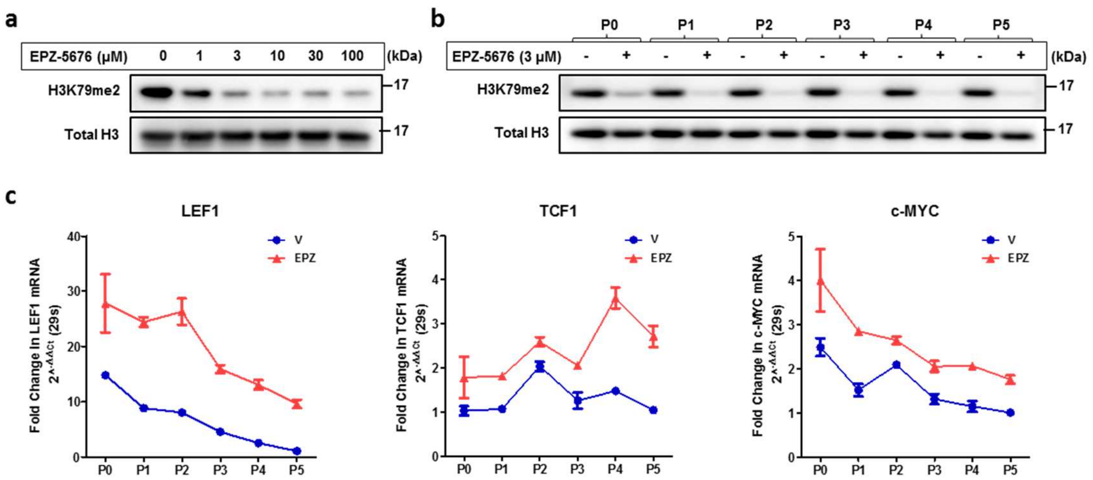


**Supplementary Figure 1 | DOT1L inhibition in a chondrocyte dedifferentiation assay.** (**a**,**b**) Immunoblot analysis showing decreased methylated H3K79 levels (H3K79me2) in human articular chondrocytes: treated with EPZ-5676 (EPZ) at the indicated concentrations or vehicle (V) for 4 days (**a**) or treated with 3 µM EPZ from passage 0 (P0) to P5 (**b**). The image is representative of one experiment. (**c**) Messenger RNA expression levels determined by quantitative PCR for *LEF1*, *TCF1* and *c-MYC* in human articular chondrocytes from P0 until P5 treated with EPZ-5676 or vehicle. Data are from one experiment with technical triplicates. Error bars indicate mean ± s.e.m.

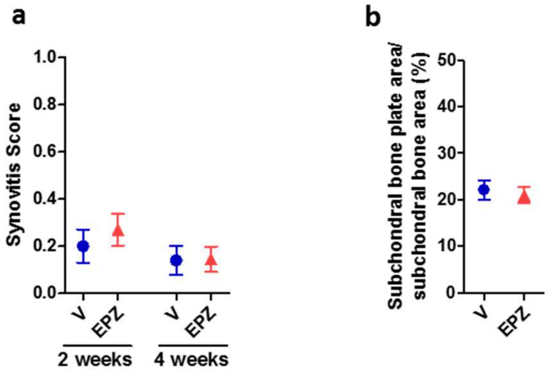


**Supplementary Figure 2 | Severity of synovitis and extent of subchondral bone remodeling in DOT1L inhibitor-injected mice.** C57/Bl6 wild-type mouse knees were injected with EPZ (5 mg/kg) or vehicle and sacrificed after 2 or 4 weeks. Knees were sectioned and stained with Hematoxylin-Safranin O. Synovitis was scored (see Methods) and is shown in (**a**)**.** Subchondral bone plate histomorphometry at the medial and lateral side of the tibia at 4 weeks was performed (see Methods) and is shown in (**b**). One experiment was performed with n=10 and 5 respectively. Error bars indicate mean ± s.e.m.

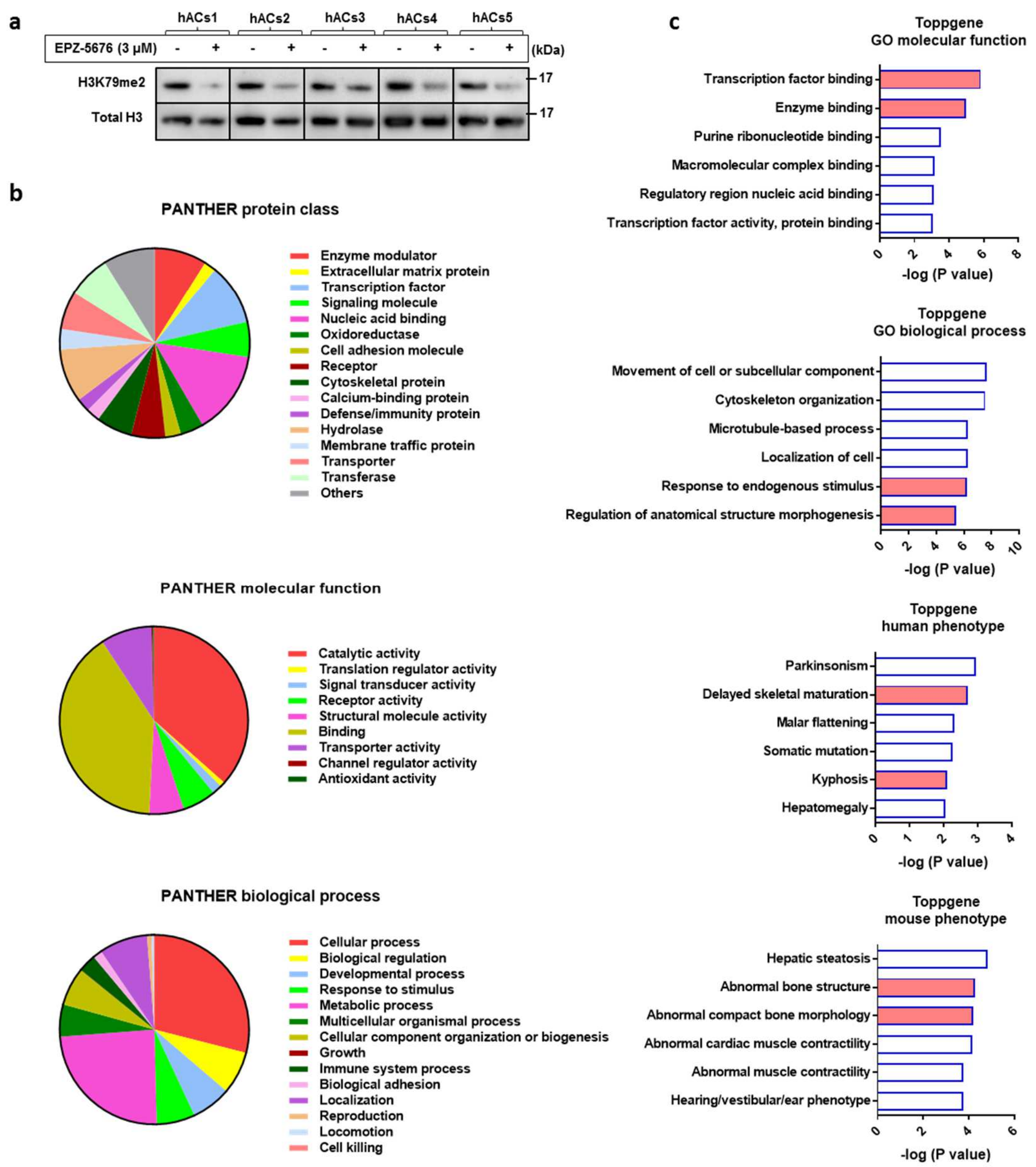


**Supplementary Figure 3 | Transcriptome network analysis of DOT1L inhibition in primary human articular chondrocytes.** (**a**) Immunoblot analysis of methylated H3K79 protein levels in 3 μM EPZ-5676 or vehicle-treated human articular chondrocytes (during 4 days) from 5 different donors (hACs1-hACs5). (**b**) Pie charts representing different categories of transcripts regulated by DOT1L using the "GO-slim" and Protein class ontology of the PANTHER Database. **(c)** Top six gene set enrichments as analysed by Toppgenesuite in the indicated categories.

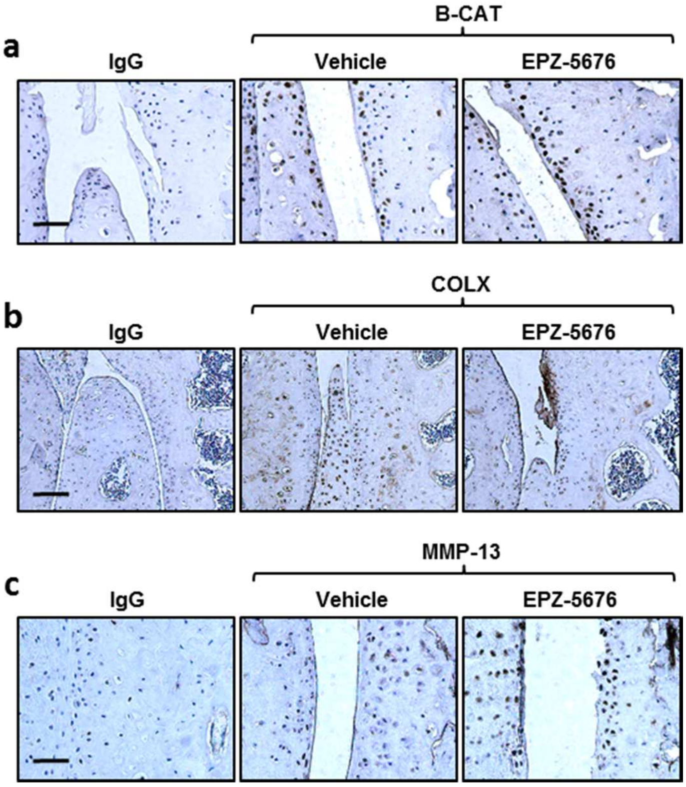


**Supplementary Figure 4 | *In vivo* DOT1L inhibition triggers articular chondrocyte hypertrophy.** (**a,b,c**) Immunohistochemistry of beta-catenin (B-CAT) (**a**), type-X Collagen (COLX) (**b**) and matrix metalloproteinase-13 (MMP-13) (**c**) in the articular cartilage of C57/Bl6 wild-type mice after injection of EPZ-5676. DOT1L inhibition did not result in detectable changes in B-CAT immunostaining. Hypertrophy markers COLX and MMP-13 are increased after EPZ-5676 treatment, particularly in the vicinity of the lesions. The images are representative of 3 different animals. Scale bar, 50 µm (**a**, **c**) or 100 µm (**b**).

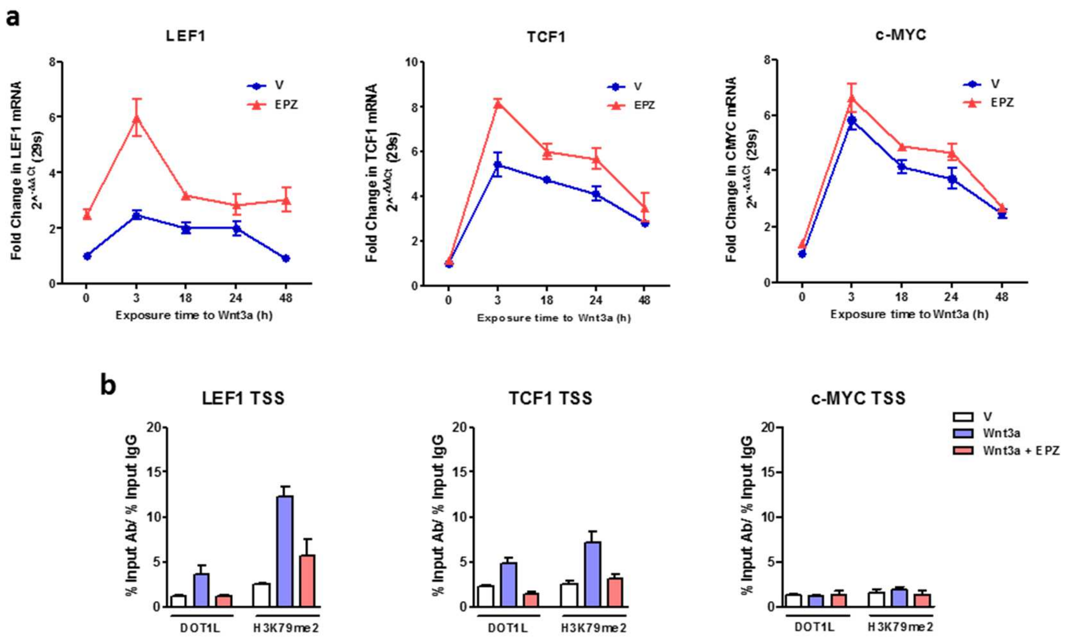


**Supplementary Figure 5 | Effects of recombinant Wnt3a on DOT1L-inhibited chondrocytes.** (**a**) *LEF1*, *TCF1* and *c-MYC* expression measured by quantitative PCR in human articular chondrocytes treated with EPZ-5676 and recombinant Wnt3a. Data are from one experiment with three technical replicates. (**b**) ChIP-qPCR analysis of DOT1L and methylated H3K79 on the transcriptional start site (TSS) of Wnt target genes. Data are from 2 experiments. Error bars indicate mean ± s.e.m.

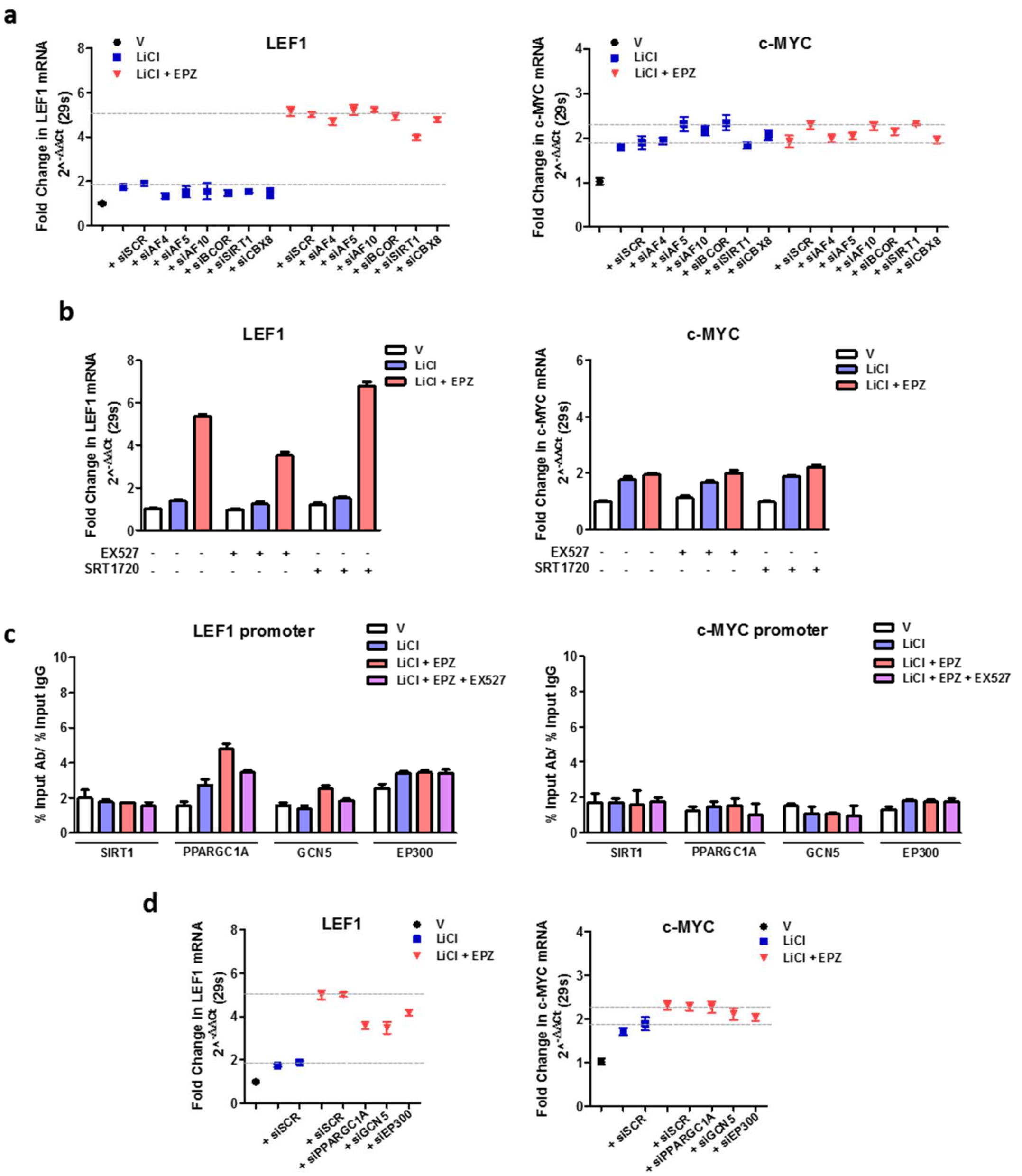


**Supplementary Figure 6 | Effects of SIRT1 and associated transcriptional activators on *LEF1* and *c-MYC* expression upon DOT1L inhibition.** All experiments were performed in healthy human articular chondrocytes, treated as indicated with DOT1L inhibitor EPZ-5676, Wnt activator LiCl, SIRT1 antagonist EX527 or SIRT1 agonist SRT1720. All data are presented as mean ± s.e.m. (**a**) Expression levels of *LEF1* and *c-MYC* Wnt target genes measured by quantitative PCR in chondrocytes transfected with indicated specific or scrambled siRNA (siSCR). Data are from one experiment with technical triplicates. (**b**) *LEF1* and *c-MYC* expression measured by quantitative PCR in the presence of a SIRT1 agonist or antagonist. Data from 2 experiments each with technical triplicates. (**c**) ChIP-qPCR analysis of SIRT1,

PPARGC1A, GCN5 and EP300 binding on the *LEF1* and *c-MYC* promoter and (**d**) *LEF1* and *c-MYC* expression after siRNA transfection with indicated specific or scrambled siRNA. Data from 2 biologically independent experiments.

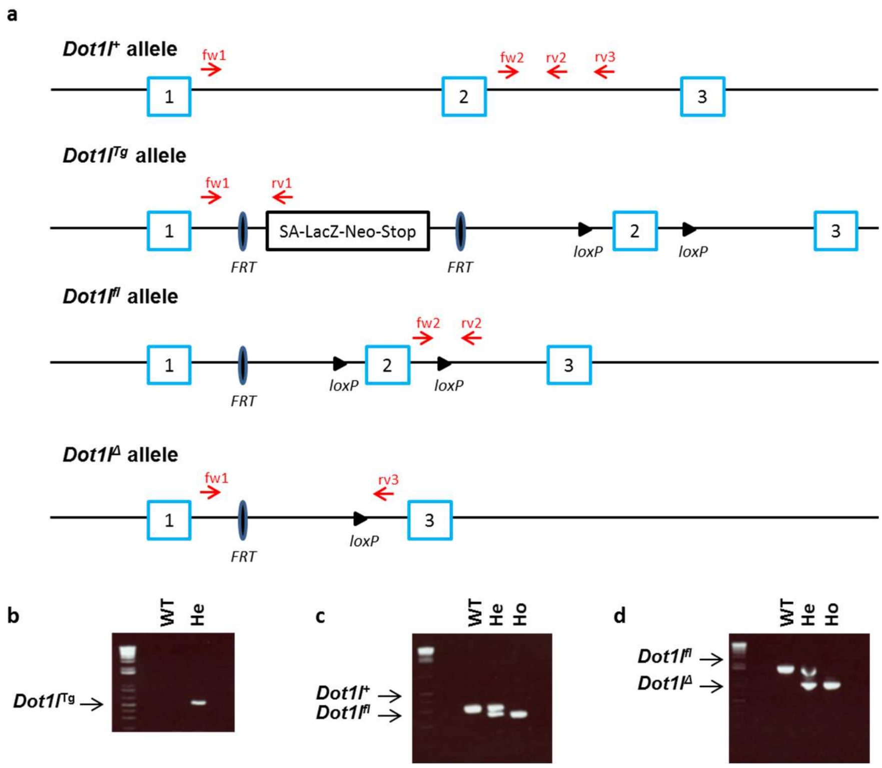


**Supplementary Figure 7 | Generation of cartilage-specific *Dot1l*$^{\Delta}$ knockout mice.** (**a**) *Dot1l*$^{Tg/+}$ mice (Knockout Mouse Project (KOMP) (CSD29070)) were crossed with FLP-transgenic mice to obtain floxed Dot1l mice (*Dot1l*$^{fl/+}$). These mice were bred to *Col2*-Cre mice to generate conditional cartilage-specific *Dot1l*$^{\Delta}$ knockout mice. Loss of exon 2 (*Dot1l*$^{\Delta/\Delta}$) led to a non-functional frame shift mutation. (**b-d**) Genotyping of *Dot1l*$^{+}$, *Dot1l*$^{Tg}$, *Dot1l*$^{fl}$ and *Dot1l*$^{\Delta}$ knockout mice. Loss of exon 2 (*Dot1l*$^{\Delta/\Delta}$) led to a non-functional frame shift mutation. (**b-d**) Genotyping of *Dot1l*$^{+}$, *Dot1l*$^{Tg}$, *Dot1l*$^{fl}$ and *Dot1l*$^{\Delta}$ alleles. (WT: wildtype, He: heterozygous, Ho: homozygous knockout).

(fw1: GAAGACTGTCTAGAGACTTAGGGTTGTGGGGCA,
rv1: CCAACTGACCTTGGGCAAGAACAT pair: *Dot1l*$^{Tg}$ (503bp);
fw2: GAGGCATCTCTCCATGACCCTTGTCTGGGAA,
rv2: AGGAACCACAGGATGCTTCA pair: *Dot1l*$^{+}$ (313bp) and *Dot1l*$^{fl}$ (265bp);
fw1, rv3: AAGGGTGTGGGTGGGCCTTCTTAGGTTAGGTATGT pair: *Dot1l*$^{fl}$ (1524bp) and *Dot1l*$^{\Delta}$ (841bp)).

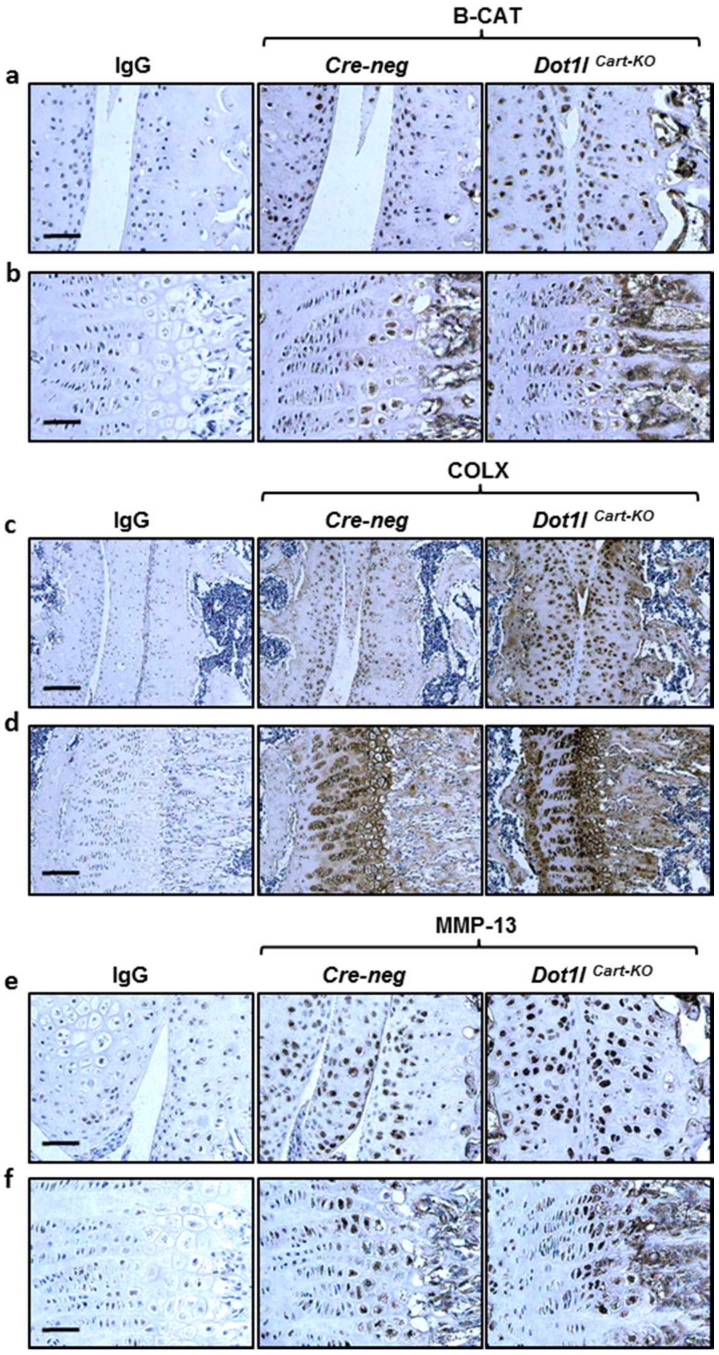


**Supplementary Figure 8 | Loss of DOT1L accelerates chondrocyte hypertrophy in cartilage-specific *Dot1l* knockout mice.** (**a-f**) Immunohistochemistry of B-CAT (**a**,**b**), COLX (**c**,**d**) and MMP13 (**e**,**f**) in the articular cartilage (**a**,**c**,**e**) and the growth plate (**b**,**d**,**f**) of 4-week-old *Cre-neg* and $Dot1l^{Cart\text{-}KO}$ mice. DOT1L loss did not result in detectable changes in B-CAT immunostaining. Hypertrophy markers COLX and MMP-13 are increased in $Dot1l^{Cart\text{-}KO}$ mice. The images are representative of 3 different animals. Scale bar, 50 µm (**a**,**b**,**e**,**f**) or 100 µm (**c**,**d**).

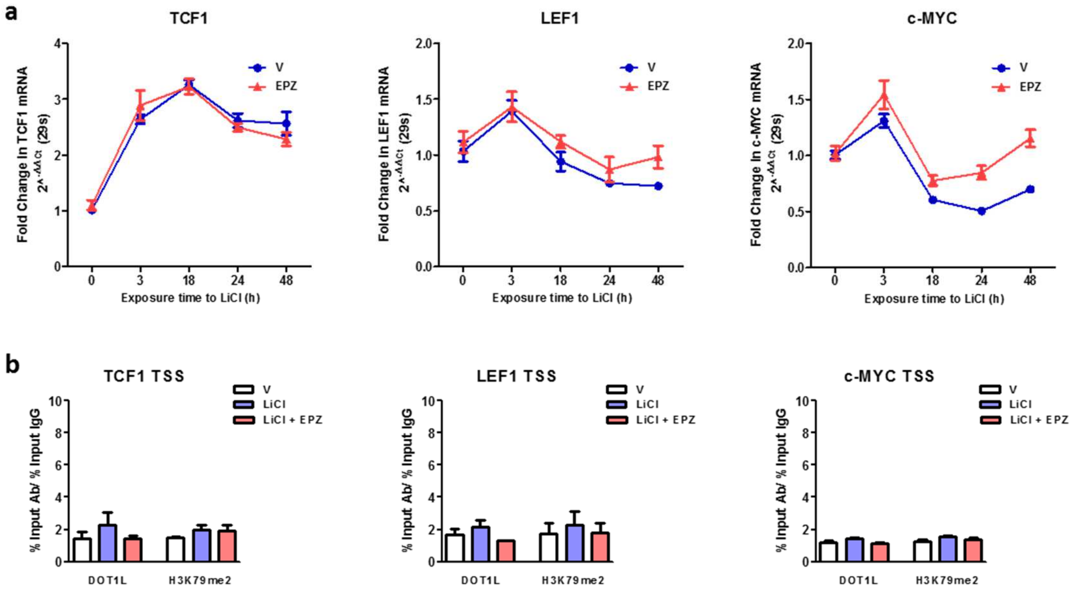


**Supplementary Figure 9 | The effect of DOT1L inhibition on Wnt target genes is absent in osteoblasts.** (**a**) *LEF1*, *TCF1* and *c-MYC* expression measured by quantitative PCR in human osteoblasts treated with EPZ-5676 and LiCl. Data are from one experiment with three technical replicates. (**b**) ChIP-qPCR analysis of DOT1L and methylated H3K79 on the transcriptional start site (TSS) of Wnt target genes in osteoblasts. Data are from two biologically independent experiments. Error bars indicate mean ± s.e.m.

## Uncropped blots Fig. 1c

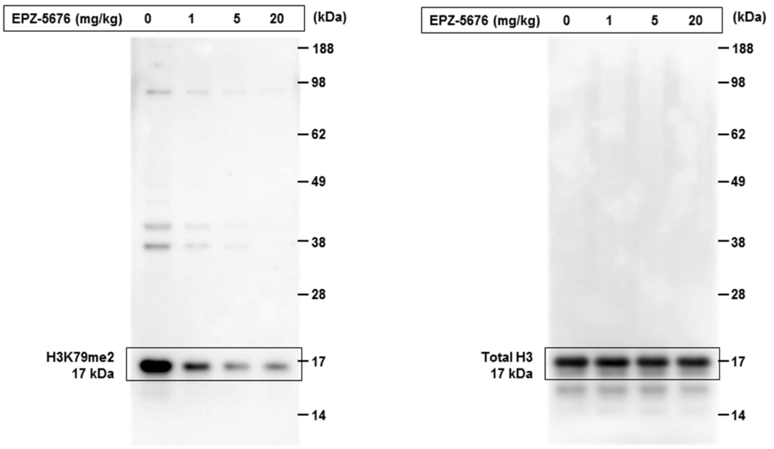


## Uncropped blots Fig. 2b

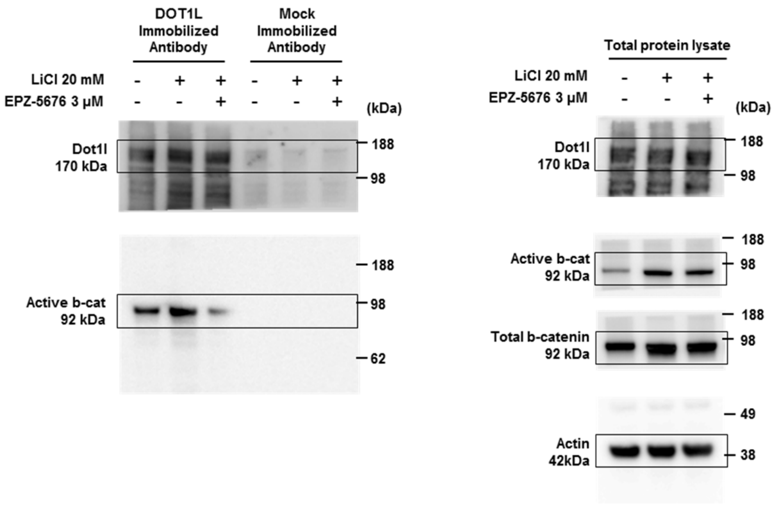

# Uncropped blots Fig. 4e

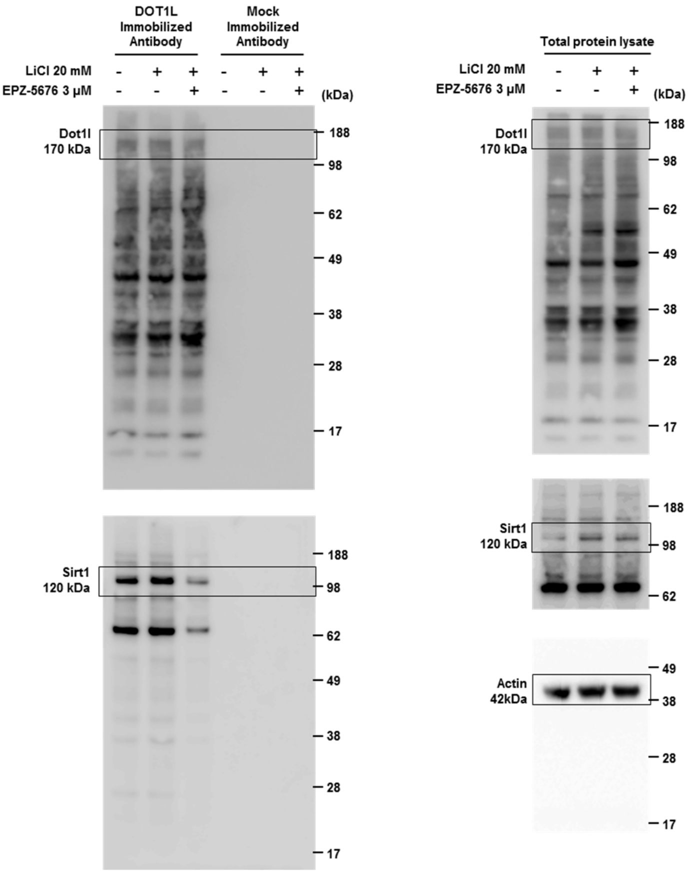


# Uncropped blots Supplementary Fig. 1

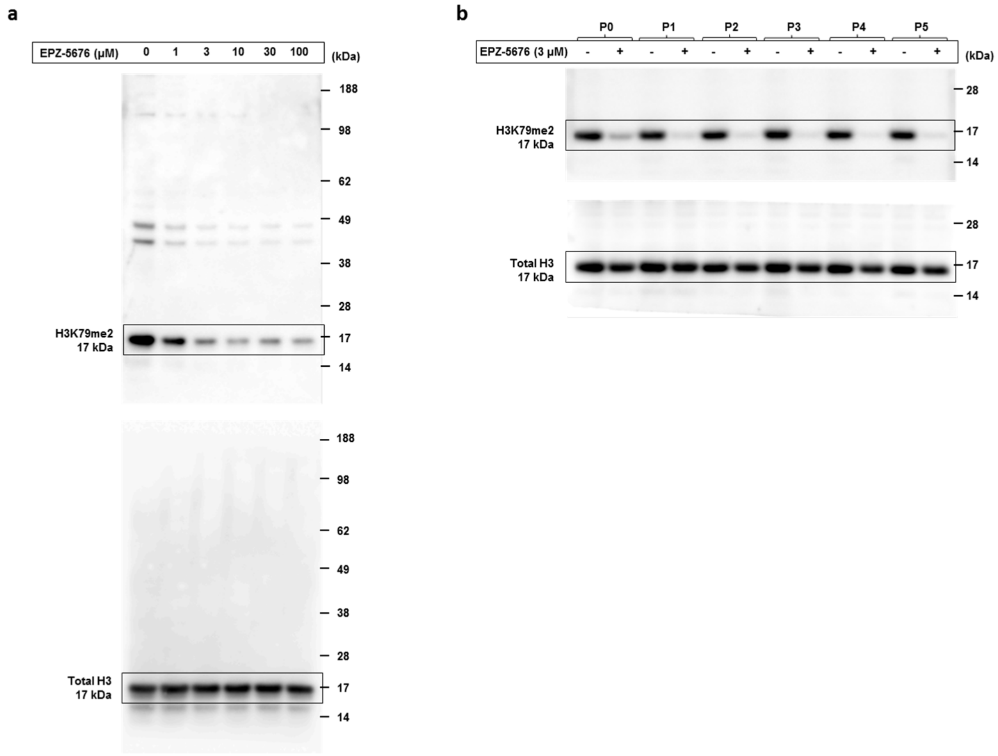

## Uncropped blots Supplementary Fig. 3a

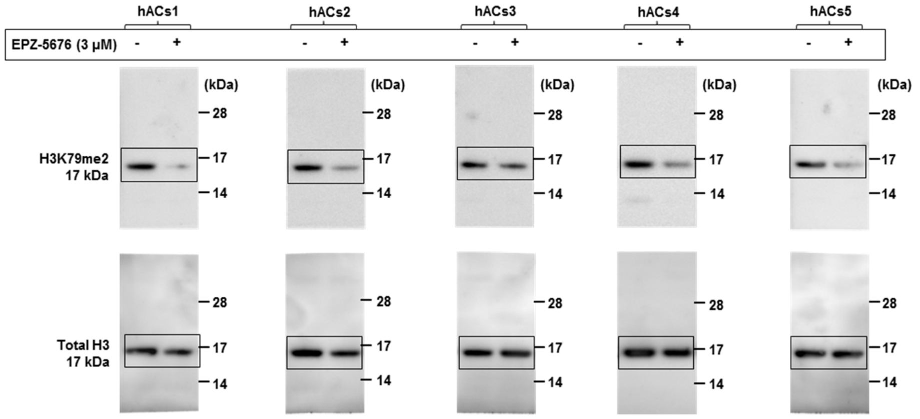


**Supplementary Figure 10 | Unprocessed original scans.** Uncropped images of scanned western blots and gels shown in the Figures are provided.

**Supplementary Table 1 | Top down- and upregulated genes upon DOT1L inhibition that were previously linked with cartilage biology.**

| Gene | Biological relevance | Log ratio microarray |
|---|---|---|
| ***Top downregulated*** | | |
| ***RGS5*** | Chondrocyte differentiation | -0,54782278 |
| ***COL6A1*** | KO mice develop osteoarthritis | -0,541920087 |
| ***SCUBE3*** | TGFβ receptor ligand | -0,476171007 |
| ***FHL1*** | Downstream target of Wnt | -0,400055228 |
| ***GDF10*** | Bmp family member | -0,392016034 |
| ***LOXL2*** | Collagen formation, chondrocyte differentiation | -0,372004916 |
| ***COL14A1*** | Plays an adhesive role by integrating collagen bundles | -0,308844443 |
| ***ACAN*** | Extracellular matrix | -0,305167395 |
| ***IGFBP5*** | IGF pathway | -0,304701203 |
| ***Top upregulated*** | | |
| **CCL7** | Upregulated in osteoarthritis | 1,038689032 |
| **CCL8** | Upregulated in osteoarthritis | 0,796655267 |
| **MMP1** | Degradation of extracellular matrix proteins | 0,65190844 |
| **DKK1** | Wnt modulator | 0,573072224 |
| **SFRP1** | Wnt modulator | 0,570064988 |
| **CTHRC1** | Inhibit collagen matrix synthesis | 0,56757143 |
| **IGF1** | IGF pathway | 0,516760007 |
| **EMILIN2** | Wnt modulator | 0,41962033 |
| **LEF1** | Wnt direct target gene | 0,404441205 |
| **GPNMB** | New gene associated with osteoarthritis | 0,32814179 |
| **DACT1** | Wnt antagonist | 0,309393924 |

**Supplementary Table 2 | siRNA catalog numbers.**

| siRNA | Catalog number |
|---|---|
| **siAF4** | Qiagen, S104147437 |
| **siAF5** | Qiagen, S104203101 |
| **siAF10** | Qiagen, S100066444 |
| **siBCOR** | Qiagen, S100311423 |
| **siCBX8** | Qiagen, S100339255 |
| **siDOT1L** | Invitrogen, AM51331 |
| **siGCN5** | Qiagen, S100426118 |
| **siP300** | Qiagen, S102622592 |
| **siPPARGC1A** | Qiagen, S100101024 |
| **siSCR** | Qiagen, S103650318 |
| **siSIRT1** | Qiagen, S100098434 |

**Supplementary Table 3 | Human primers used for qPCR.**

| Primer name | Sequence |
|---|---|
| **ACAN-Fw** | ACAAGGTCTCACTGCCCAAC |
| **ACAN-Rv** | AATGGAACACGATGCCTGTC |
| **CCL7-Fw** | ATGAAAGCCTCTGCAGCACT |
| **CCL7-Rv** | CCAGCCTCTGCTTAGGGATT |
| **CCL8-Fw** | GTTTCTGCAGCGCTTCTGTG |
| **CCL8-Rv** | CACGTTAAAGCAGCAGGTGA |
| **CHI3L2-Fw** | CCCTTATCACTGGCCACAAC |
| **CHI3L2-Rv** | CCACCTTCTCTGATGGCATT |
| **c-MYC-Fw** | TTTCGGGTAGTGGAAAACCA |
| **c-MYC-Rv** | GCTCGAATTTCTTCCAGATATCCTC |
| **COL1A1-Fw** | AAGAGGAAGGCCAAGTCGAG |
| **COL1A1-Rv** | CACACGTCTCGGTCATGGTA |
| **COL2A1-Fw** | GCACCTGCAGAGACCTGAAA |
| **COL2A1-Rv** | GTCTCGCCAGTCTCCATGTT |
| **COL6A1-Fw** | ATCGACAACCTGAGGGACAG |
| **COL6A1-Rv** | TGATAGCGCAGTCGGTGTAG |
| **COL14A1-Fw** | TTCAAGGCCTTATGCCAGAC |
| **COL14A1-Rv** | ACTCTGGGCTTTGGATCCTT |
| **CTHRC1-Fw** | TCGAGCGCCTCTGAGATTAT |
| **CTHRC1-Rv** | CTTTGAATCCATCCCGACCT |
| **DACT1-Fw** | AGTCGCCTGGAGGAGAAGTT |
| **DACT1-Rv** | GCTCTTGCAACTGATTCAACAA |
| **DKK1-Fw** | CGGGAATTACTGCAAAAATGG |
| **DKK1-Rv** | ACCCATCCAAGGTGCTATGA |
| **EDNRA-Fw** | AATGGCAGCTTGAGAATTGC |
| **EDNRA-Rv** | TGAAGAGGGAACCAGCAAAG |
| **EMILIN2-Fw** | GGCTTTAGAGGGGGAGATTG |
| **EMILIN2-Rv** | TTGGGGACAAAGTCTTCCTG |
| **FHL1-Fw** | TGGCTCTGGAGCTAATTTGG |
| **FHL1-Rv** | GGCCATCCTTTTGCACATAC |
| **FRZB-Fw** | GAGGAGCTGCCAGTGTACGA |
| **FRZB-Rv** | ACTGCTTGCCCCTCTACAGTT |
| **GDF10-Fw** | GTCCACATGCACAGGCTCTAT |
| **GDF10-Rv** | TGCATGGAAGTCAGGTTGAA |
| **GPNMB-Fw** | TTCCTGGGATTTCTGCTCCT |
| **GPNMB-Rv** | TGTGCTCCCTCATGTAAGCA |
| **HEY2-Fw** | TGGGGAGCGAGAACAATTAC |
| **HEY2-Rv** | CCTCTCCTTTTCTTTCTTGCCATA |
| **IGF1-Fw** | CAGCAGTCTTCCAACCCAAT |

| | |
|---|---|
| **IGF1-Rv** | ACAGCGCCAGGTAGAAGAGA |
| **IGFBP5-Fw** | GGTTTGCCTCAACGAAAAGA |
| **IGFBP5-Rv** | AGTAGGTCTCCTCGGCCATC |
| **ITGB3-Fw** | GTGTGCCTGGTGCTCTGATG |
| **ITGB3-Rv** | ACTCACTGGGAACTCGATGG |
| **LEF1-Fw** | CAGTCATCCCGAAGAGGAAG |
| **LEF1-Rv** | AGGGCTCCTGAGAGGTTTGT |
| **LOXL2-Fw** | CCTGGGGAGAGGACATACAAT |
| **LOXL2-Rv** | AGCTTGCAGCTGGAGATGTG |
| **MATN3-Fw** | GACAGAACAGGGTCCCATCA |
| **MATN3-Rv** | TGAGAGCCTAGGGCACACTT |
| **MMP1-Fw** | TTCGGGGAGAAGTGATGTTC |
| **MMP1-Rv** | GTCGGCAAATTCGTAAGCAG |
| **MMP3-Fw** | GGCCAGGGATTAATGGAGAT |
| **MMP3-Rv** | TGAAAGAGACCCAGGGAGTG |
| **NOTCH3-Fw** | GTGTGCAAATGGAGGTCGTT |
| **NOTCH3-Rv** | CCACTGAACTCTGGCAGACA |
| **RGS5-Fw** | TCAGTTGGTGACCTTGTCATTC |
| **RGS5-Rv** | TTGTTCTGCAGGAGTTTGTCC |
| **RIPK4-Fw** | CTTCGGGCAGGTGTACAAGG |
| **RIPK4-Rv** | TTCTTCCAAAAGCTCCATGC |
| **S29-Fw** | CCAGCAGCTCTACTGGAGTCA |
| **S29-Rv** | GCCTATGTCCTTCGCGTACT |
| **S100B-Fw** | AGGGAGACAAGCACAAGCTG |
| **S100B-Rv** | GAAGTCACATTCGCCGTCTC |
| **SCUBE3-Fw** | CGGACACAACTGTCTGGATG |
| **SCUBE3-Rv** | GTTGTCGCTGAGGAAGAAGC |
| **SERPINA1-Fw** | GGGAAACTACAGCACCTGGA |
| **SERPINA1-Rv** | CCCCATTGCTGAAGACCTTA |
| **SFRP1-Fw** | CAAGCCCCAAGGCACAAC |
| **SFRP1-Rv** | TTCATCCTCAGTGCAAACTCG |
| **SLIT3-Fw** | CCAGGATCACCAAGATGGAC |
| **SLIT3-Rv** | CGCTCTAGCTGCTTCAGGTC |
| **SOX9-Fw** | GGTGCTCAAAGGCTACGACT |
| **SOX9-Rv** | GTAATCCGGGTGTCCTTCT |
| **TCF1-Fw** | CACCCATCCTCAAAGAGCTG |
| **TCF1-Rv** | ATGTTGTGCTGCTGCAGGTA |

**Supplementary Table 4 | Human primers used for ChIP-qPCR.**

| Primer name | Sequence |
|---|---|
| **c-MYCpromoter-Fw** | CCGCCTGCGATGATTTATAC |
| **c-MYCpromoter-Rv** | GCTCCCTCTCAAACCCTCTC |
| **c-MYC-TSS-Fw** | GGCACTTTGCACTGGAACTT |
| **c-MYC-TSS-Rv** | CTTTCAGAGAAGCGGGTCCT |
| **LEF1promoter-Fw** | AGCAATTGGCAGCCCTATTT |
| **LEF1promoter-Rv** | TGCAAACCAGTCTGCTGAAC |
| **LEF1-TSS-Fw** | AGAAAAACCGAAGCGAAAGG |
| **LEF1-TSS-Rv** | AACGAAACGTCCACTTCCTG |
| **TCF1promoter-Fw** | AGTAAGCGGGGTCAGGAGTT |
| **TCF1promoter-Rv** | CCACTCGGCATAGCCTTAAA |
| **TCF1TSS-Fw** | AGCTCGCGGAGCCGCTCTGC |
| **TCF1-TSS-Rv** | GGGGCGCGGGGCGCTGGGCG |
